# Leveraging real-world data for safety signal detection and risk management in pre- and post-market settings

**DOI:** 10.3389/fdsfr.2025.1626822

**Published:** 2025-10-17

**Authors:** Kathleen M. Gavin, Matthew L. Sundermann, Alethea Wieland

**Affiliations:** 1 Datavant, Phoenix, AZ, United States; 2 Advarra, Columbia, MD, United States

**Keywords:** real world data, safety surveillance, pharmacovigilance, risk management, privacy preserving record linkage (PPRL), tokenization, data linkage, federated data sharing

## Abstract

The evolving regulatory landscape has increasingly recognized the value of real-world data (RWD) in enhancing drug safety surveillance across the clinical development lifecycle. Enabled by frameworks such as the FDA’s Real-World Evidence (RWE) Programs and other international regulatory bodies, sponsors now have expanded opportunities to use RWD to detect, evaluate, and manage safety signals in both pre- and post-market settings. This paper examines how the integration of RWD, particularly through privacy-preserving record linkage (PPRL) methods like tokenization, can improve pharmacovigilance by enabling longitudinal safety monitoring while protecting patient privacy. Traditional safety surveillance methods, such as spontaneous adverse event reporting and aggregate signal detection, are limited by under-reporting and fragmented data sources. In contrast, linked RWD offers more comprehensive, patient-level insights into safety outcomes, including rare events, off-label use, and long-term risks. The paper outlines regulatory considerations for using de-identified, linked RWD in safety reporting, emphasizing the importance of clear protocols, IRB engagement, and legal compliance with HIPAA. It further highlights emerging best practices for integrating RWD into clinical development, such as early regulatory engagement and the incorporation of linked RWD-derived safety signals into risk management plans. Ultimately, we propose that leveraging linked RWD in a privacy-focused manner enables more proactive, scalable, and effective pharmacovigilance. This approach supports earlier detection of safety issues, enhances post-market follow-up, and promotes data continuity between trial and real-world settings, positioning RWD as a cornerstone of modern safety surveillance.

## 1 Introduction

In 2016, the 21st Century Cures Act was signed into law with the intent to accelerate medical product innovation and development (Congress, 2016). This enactment significantly expanded the role of Real-World Data (RWD) in regulatory decision-making by directing the U.S. Food and Drug Administration (FDA) to develop frameworks for its use in evaluating the safety and effectiveness of medical products. This legislation reflected on prior decades of progress made toward the incorporation of RWD in both the approval of new drug indications and in post-market surveillance, thereby enhancing the FDA’s ability to monitor and assess drugs in real-world settings.

Since 2016, the FDA has developed a comprehensive framework (U.S. Food and Drug Administration, 2018a) and issued several guidances (U.S. Food and Drug Administration, 2023a; U.S. Food and Drug Administration, 2023b; U.S. Food and Drug Administration, 2024; U.S. Food and Drug Administration, 2018b; U.S. Food and Drug Administration, 2023c; U.S. Food and Drug Administration, 2022; U.S. Food and Drug Administration, 2017) to help clinical development teams effectively utilize RWD and Real-World Evidence (RWE) in regulatory submissions. These resources focus on using RWD for both safety and efficacy assessments of both investigational and approved medical products, whether through pre-planned analyses in pre-market studies or ongoing post-market surveillance, ensuring that these data sources meet regulatory standards for relevance and reliability.

Other regulatory bodies including Australia’s Therapeutic Goods Administration (TGA), China’s National Medical Products Administration (NMPA), the European Medicines Agency (EMA), Health Canada (HC), Japan’s Pharmaceuticals and Medical Devices Agency (PMDA), and the United Kingdom’s (U.K.) Medicines and Healthcare Products Regulatory Agency (MHRA), have established their own principles or guidance for adopting RWD and RWE for potential regulatory decision-making and post-market surveillance (Duke- Margolis Institute for Health Policy, 2025). Representing a culmination of the Heads of Medicines Agency (HMA)-EMA Big Data Steering Group work plan efforts, the EMA fully operationalized the Data Analysis and Real World Interrogation Network (DARWIN EU^®^) in 2024 including a regionalized database and coordinating center, with plans to gain more data partners over time (European Medicines Agency, 2025). U.S. and international interest in utilizing RWD as part of regulatory activities continue to grow exponentially.

This paper explores the current opportunities and challenges associated with broadening the use of RWD, including incorporation of data linkage, for safety signal detection and risk management in both pre- and post-market settings, with a focus on the United States (U.S.). It also delves into the evolving methodologies that support the use of RWD, highlighting the potential for improved patient outcomes through more responsive and comprehensive pharmacovigilance practices.

## 2 FDA RWD framework and programs

In 2018, FDA released its Real-World Evidence Framework, as mandated by the 21st Century Cures Act (U.S. Food and Drug Administration, 2018a), which focused on using RWD to generate RWE in areas such as: supporting approvals for new drug indications, monitoring post-market drug safety and effectiveness, and guiding the development of regulatory standards for the use of RWD in various stages of clinical development. In the framework, FDA defines RWD as “data relating to patient health status and/or the delivery of healthcare routinely collected from a variety of sources”. Furthermore, the Agency defines RWE as the “clinical evidence about the usage and potential benefits or risks of a medical product derived from analysis of RWD”.

Recently published guidance documents have covered RWD data standards and how electronic health records (EHR) and claims data are best assessed for regulatory decision-making (U.S. Food and Drug Administration, 2018a; U.S. Food and Drug Administration, 2023b; U.S. Food and Drug Administration, 2024). In addition, program specific disease areas such as oncology have been developed to meet disease specific RWE needs (U.S. Food and Drug Administration, 2025).

A common theme among guidance documents is the focus on the relevance, reliability, and traceability of RWD. However, it is well recognized that all the data necessary for an individual is likely not available from one data source. In its July 2024 guidance document regarding the use of EHR and medical claims data to support regulatory decision-making, FDA recognized the need for data linkage techniques and privacy-preserving methods to enable the combination of RWD from different sources while maintaining patient confidentiality (U.S. Food and Drug Administration, 2024). As such, in recent years, RWD and RWE, including the utilization of data linkage, have increasingly been in the spotlight (Beaulieu-Jones et al., 2020; Dang, 2023; Khosla et al., 2018; Tyagi and Willis, 2025; Fitzpatrick et al., 2018).

When considering the historical use of RWD and RWE in FDA-supported activities, monitoring post-market drug safety has been the primary use case. De-identified datasets are regularly assessed in aggregate to find safety signals of interest. Enhancements in privacy preserving record linkage (PPRL) technologies have afforded the opportunity to link disparate datasets, including pre-market clinical trial data with RWD at the individual level, advancing its potential utility in support of approvals for new drugs and indications. In this paper, we will highlight how data linkage technology has advanced the usage of RWD in each of those settings.

## 3 Pharmacovigilance, safety surveillance and risk management

### 3.1 Post-market requirements for safety surveillance

At the time of medical product authorization, there are known limitations of the safety profile of a medical product. Pre-market clinical trials are often underpowered to find medical product safety issues that may be increasingly rare and oftentimes do not include patient populations and settings that reflect clinical care. To account for these limitations, post-marketing pharmacovigilance and medical device vigilance have been performed through safety surveillance strategies including spontaneous adverse event (AE) reporting and signal detection methodologies. However, under-reporting is a factor post-market, based upon a highly variable but low “voluntary” reported number of cases amongst prescribing users, patients, or caregivers themselves (Dhodapkar et al., 2022).

The use of RWD and RWE is common in post-market safety surveillance (Lavertu et al., 2021). The implementation of a post-market RWE strategy helps address the gaps that a pre-market clinical trial may have missed. Furthermore, RWE may also contain data from patient populations and settings that may not be captured in a clinical study, such as off-label use at a physician’s discretion (TransCelerate BioPharma Inc., 2025). In these cases, large de-identified datasets can be used for aggregate reporting of safety signals.

#### 3.1.1 Spontaneous adverse event reporting

Spontaneous AE reporting involves the unsolicited communication of suspected adverse drug reactions (ADRs) from healthcare professionals, patients, or consumers to regulatory authorities, pharmaceutical companies, or other relevant bodies. Spontaneous reporting is essential for identifying new safety signals, previously unknown or poorly characterized ADRs, that may not have been detected in pre-market clinical trials due to their limited size, duration, or population scope. Regulatory agencies like FDA in the U.S. and EMA in Europe analyze these reports to monitor the safety of marketed drugs. When a potential safety signal is identified, it may lead to further investigation, regulatory action, or communication efforts to inform healthcare providers and the public.

Combining spontaneous reporting data with RWD could create a more comprehensive safety surveillance system. For example, signals from spontaneous reports could trigger targeted RWD analyses to validate or refine the signal. Also, real-time RWD sources (e.g., EHR systems) could evolve into active surveillance tools, identifying and reporting potential safety signals alongside traditional spontaneous systems.

#### 3.1.2 Safety signal assessment

Safety signal assessment or signal management is a second type of safety surveillance. It is a component of modern pharmacovigilance that comprises an analysis of information that may arise from a variety of healthcare data sources (CIOMS, 2010). Using methods such as disproportionality analysis, data mining, and statistical modeling, safety surveillance systems can detect patterns in the reported adverse events that may indicate a possible safety concern. The goal of signal management is to evaluate any information that may suggest a causal association or a new development of a known association between a medical product and a safety event (European Medicines Agency, 2017; Beninger, 2018; Beninger, 2020; Ibrahim et al., 2021). In this context, new evidence is commonly gathered on a medical product through the surveillance of a variety of RWD sources including EHRs, insurance claims, registries, and complaint databases among other sources. In addition to these methods, the FDA has active programs at varying levels of maturity that have established a surveillance infrastructure for active post-market risk identification and analysis using de-identified RWD such as 1) the Sentinel System for approved medical products regulated by the Center for Drug Evaluation and Research (CDER) (Sentinel Initiative, 2025), 2) the Biologics Effectiveness and Safety (BEST) Initiative, part of the Center for Biologics Evaluation and Research (CBER) surveillance program (BEST Initiative, 2025) and 3) the National Evaluation System for health Technology (NEST) for medical devices run in collaboration by the Center for Devices and Radiological Health (CDRH) and the Medical Device Innovation Consortium (MDIC) (US Food and Drug Administration, 2019).

Safety signal assessment follows a detailed process beginning with detection of a safety event that may be recorded in usual care or a hospitalization event in the EHR by a healthcare provider. Voluntarily, the healthcare provider may report the event directly to a regulatory agency or the Manufacturer (as described in Section 3.1.1). Additionally, sponsors are required to report suspected unexpected serious adverse reactions (SUSARs) and adverse events of special interest (AESIs) to regulatory agencies in a pre-market setting. The signals detected via these multiple pathways must be validated and prioritized against other signals and stakeholders must provide a recommendation of action followed by a communication of an executable plan (TransCelerate BioPharma Inc., 2025).

### 3.2 Privacy considerations

Under the Health Insurance Portability and Accountability Act (HIPAA) Privacy Rule, health information is considered de-identified when it does not identify an individual and there is no reasonable basis to believe it can be used to identify an individual (Congress, 1996; CFR, 2025a). The Rule outlines two acceptable methods for achieving de-identification. The first is the Expert Determination method, in which a qualified expert applies statistical and scientific principles to determine that the risk of re-identification is very small and documents the methodology and results (CFR, 2025a; U.S. Department of Health and Human Services Office for Civil Rights OCR, 2012). The second is the Safe Harbor method, which requires the removal of 18 specific identifiers (e.g., names, geographic details, dates directly related to an individual, and other unique identifiers) and the absence of actual knowledge that the remaining information could be used to identify the individual (CFR, 2025a; U.S. Department of Health and Human Services office for Civil Rights OCR, 2012). These de-identification standards enable the use and sharing of health data in a manner that protects patient privacy while allowing for secondary data use. Expert Determination specifically, provides an avenue for de-identified data linkage and analysis across disparate sources that can be included as part of postmarket safety surveillance activities.

However, identified safety reporting requirements are important to consider in safety surveillance. In large de-identified RWD databases, the Covered Entity is in control of all patient data and any de-identification or re-identification process required for outside third parties (CFR, 2025b). If connection back to a specific identified patient is recommended as part of the safety reporting/investigation process, it can only be performed by the Covered Entity (CFR, 2025b). For maintaining a legal privacy framework for the utilization of identified data or for re-identification needs, several mechanisms are in place under HIPAA for Preparatory to Research, Research Use/Disclosure Without Authorization, Research Use/Disclosure with Individual Authorization, Limited Datasets with Data Use Agreements, Research on Decedents, or Research Exemption determinations (CFR, 2025a; CFR, 2025c; CFR, 2025d; CFR, 2025e; CFR, 2025f; CFR, 2025g). The best resources to discuss the need for re-identification of data for safety reporting requirements is the Institutional Review Board (IRB) of Record, Privacy Officer of the Covered Entity, and/or applicable regulatory agency.

The ability to re-identify for safety reporting is also dependent upon the type of entity providing the RWD. In healthcare data privacy, data aggregators, originators, and covered entities each have distinct roles and permissions around the use of identified and de-identified data. Aggregators collect and analyze de-identified data across sources but are restricted from re-identification without authorization. Covered entities can include healthcare providers, health plans and healthcare clearinghouses (and their business associates) that transmit health information electronically in connection with a covered transaction, and generally have broad access to identified data for treatment, payment, and operations have to comply with the HIPAA Rules (CFR, 2025b). They can de-identify and commercialize their data, and depending on the use case can have the ability to perform re-identification if needed. A data originator is anyone who generates or collects patient data but are not always considered covered entities under HIPAA. This could include individuals and organizations but also devices that generate health data. Together, these roles help balance data use for research and privacy protections. The RWD repositories and infrastructure supported by the FDA as well as sponsor specific compiled real-world databases are built to comply with these privacy requirements (Rosati et al., 2018).

Privacy regulations often limit the ability to share patient level health data. Federated data sharing models are a strategy in which data partners maintain control over de-identified patient-level data and execute standardized queries locally, enhancing privacy and governance (Li et al., 2024). To support consistent analytics across distributed sources, they rely on various common data models such as the Sentinel Common Data Model, the Observational Medical Outcomes Partnership (OMOP) model, and the Patient-Centered Outcomes Research Network (PCORnet) model, with some programs also incorporating Fast Healthcare Interoperability Resources (FHIR)-based structures. The FDA infrastructure surveillance programs mentioned previously (e.g., Sentinel) utilize federated data sharing and common data models. Indeed, DARWIN EU also employs a federated approach, using the OMOP common data model to enable consistent, privacy-preserving analysis across a growing network of European data partners. This approach enables scalable, regulatory-grade evidence generation while accommodating the diverse data environments of participating institutions.

## 4 Opportunities and strategies for leveraging RWD/RWE across pre- and post-market evidence generation

The integration of linked RWD into both pre- and post-market integrated evidence generation strategies presents significant opportunities to enhance safety signal detection, evaluation, and management. While FDA has provided guidance on RWD, an evolving regulatory landscape means that stakeholders face uncertainty about how RWD and its linkage will be evaluated. This uncertainty can hinder the adoption of RWD in both pre-market clinical trials and post-marketing surveillance efforts. Current best practices involve the integration of a multi-component team, early engagement with regulators, and clearly written protocols that include data sources to be used, proposed methodology, and the planned reporting structure (see Supplementary Material for additional details on best practices), although additional guidance from regulators and real-world case studies are still needed.

### 4.1 Beyond single RWD sources: the value of linking data

Historically, researchers often had to rely on summary-level aggregate analyses of RWD due to concerns over patient privacy, data security, and the complexities of managing identified data, lacking the granularity to understand patient-specific factors or to track events longitudinally. Individual level data linkage is a growing opportunity across the clinical development lifecycle as it provides the opportunity to integrate data across disparate sources at the patient level. In pharmacovigilance and safety surveillance, linkage enables a more complete and longitudinal picture of patient experiences by providing the opportunity to connect medication exposures, clinical outcomes, and healthcare utilization across differing points of care and data sources. This may allow for earlier identification of rare or unexpected safety signals, better differentiation between drug-related and background incidence, and the ability to monitor long-term or delayed safety outcomes that may not surface in clinical trials alone. By capturing data from diverse sources, linkage also supports subgroup analyses to understand differential risks in vulnerable populations, ultimately contributing to more robust regulatory decision-making and patient safety.

Several approaches can be used to match individual level health data for reliable linkage (Ong et al., 2020; Eisinger-Mathason et al., 2025). Deterministic matching relies on exact matches of unique identifiers (e.g., name, date of birth, national identifier) and is highly precise but limited by data quality and availability of identifiers. Probabilistic matching uses statistical methods to calculate the likelihood of a match based on one or more attributes, making it useful when some identifiers are missing or slightly varied but requiring more computation and validation. Referential matching leverages external reference databases to resolve changes or differences in identifiers and improve match accuracy, often in combination with probabilistic approaches. Together, these methods provide flexible options depending on regulatory, clinical, and data contexts.

A recent review found more than 70 articles published between 2016 and 2023 using linkage of clinical trials to routinely collected data for a variety of use cases, with almost all relying on direct patient identifiers (e.g., UK National Health Service (NHS) number, Medicare number, or US Social Security number) (NajafZadeh et al., 2025). However, reliance on direct identifiers for patient matching in long-term pharmacovigilance is limited by privacy risks, regulatory constraints, and data quality issues, making privacy-preserving methods a more scalable and compliant alternative. The advent of PPRL, as described in more detail below, addresses many of these limitations, enabling secure and de-identified data integration while maintaining compliance with privacy regulations (Eisinger-Mathason et al., 2025).

Ultimately, linking existing RWD has the potential to significantly reduce the reliance on costly, investigator-led studies for post-market surveillance, offering a more resource-efficient solution for long-term safety monitoring. Table 1 presents the key considerations for utilizing RWD as well as the added benefit of data linkage in safety surveillance.

**TABLE 1:** Key considerations for utilizing RWD and added benefits of data linkage in safety surveillance.

Use case	Challenge	RWD consideration	Added value of linkage	Overall impact
**The research or surveillance application**	The difficulty of answering the question without linked data	What a single dataset can (or cannot) offer in this use case	What becomes possible only by combining/linking multiple datasets	The higher-level outcome
**Adverse Drug Reactions (ADRs)**	Sporadic ADRs occur across multiple healthcare settings, making causality and incidence difficult to assess	A single data source (e.g., EHRs) may capture drug exposure or outcomes, but not both comprehensively	Linking medical claims, EHRs, and pharmacy data fills gaps across sources to create a more complete safety profile	Enables timely detection of new or rare ADRs and improves the accuracy of risk assessments
**Long-Term Safety Outcomes**	Events like organ toxicity or chronic cardiovascular risks may appear years after drug initiation	Individual datasets often lack longitudinal follow-up, leading to gaps over time or across care systems	Patient-level linkage across claims, EHRs, and registries supports long-term, geographically broad monitoring	Facilitates the study of delayed or chronic adverse effects, essential for therapies like biologics or gene therapies with extended post-market obligations
**Subpopulation-Specific Safety Analysis**	Vulnerable subgroups (e.g., elderly, pregnant individuals, comorbid patients) are underrepresented or fragmented across data	A single dataset may capture demographics or outcomes, but rarely both with sufficient depth	Linking datasets allows stratified analyses by combining demographic, treatment, and outcome information	Improves understanding of differential drug effects and safety profiles across diverse populations
**Rare Event Detection**	Individual datasets lack sufficient size to detect rare events	One source alone may miss signals or suffer from underreporting	Aggregating across multiple datasets increases sample size and statistical power	Enables earlier identification of rare but serious safety signals
**Off-Label Use**	Off-label prescribing is poorly captured in trial and postmarket data and often underreported	Claims or prescribing records show patterns, but don’t link directly to outcomes	Combining prescribing/claims with outcomes data reveals both use patterns and associated risks	Supports proactive monitoring and targeted education around off-label use
**Comparative Safety Analysis**	Difficult to compare drug safety without integrated data covering comparable cohorts	A single dataset may lack appropriate comparator populations or granular enough detail to develop appropriately matched cohorts	Linked datasets enable matched cohort creation and robust comparisons across drugs	Provides robust comparative safety profiles, aiding regulatory and payer decision-making

### 4.2 Privacy-preserving record linkage

PPRL refers to a set of techniques that enable secure, patient-level linkage of health data across datasets without exposing personally identifiable information (PII). One common approach is tokenization, where PII elements (e.g., name, date of birth) are irreversibly hashed and then encrypted in a site-specific manner to generate persistent, de-identified tokens. These tokens allow records from different organizations to be matched at the individual level without revealing the identity of that individual. The matching methodologies described above (e.g., deterministic, probabilistic, referential) also apply to PPRL, only using encrypted hashes/codes to represent underlying identifiers instead of utilizing in their original raw format. Commercial vendors such as Datavant and HealthVerity provide proprietary PPRL infrastructures, while open-source methods, including PPRL (R-based) (CRAN, 2022), clkhash/Anonlink (Python-based) (Clkhash, 2019), and PRIMAT (Java-based) (Github, 2025), offer public alternatives. The use of tokenization is particularly of interest in the US, where fragmented care delivery and varied healthcare coverage (including the lack of a national health ID) make it difficult to follow patients across systems. By converting identifiers into privacy-preserving tokens, it enables secure linkage of records across disparate sources, protecting patient privacy while creating a more complete view of healthcare experiences.

**FIGURE 1:**
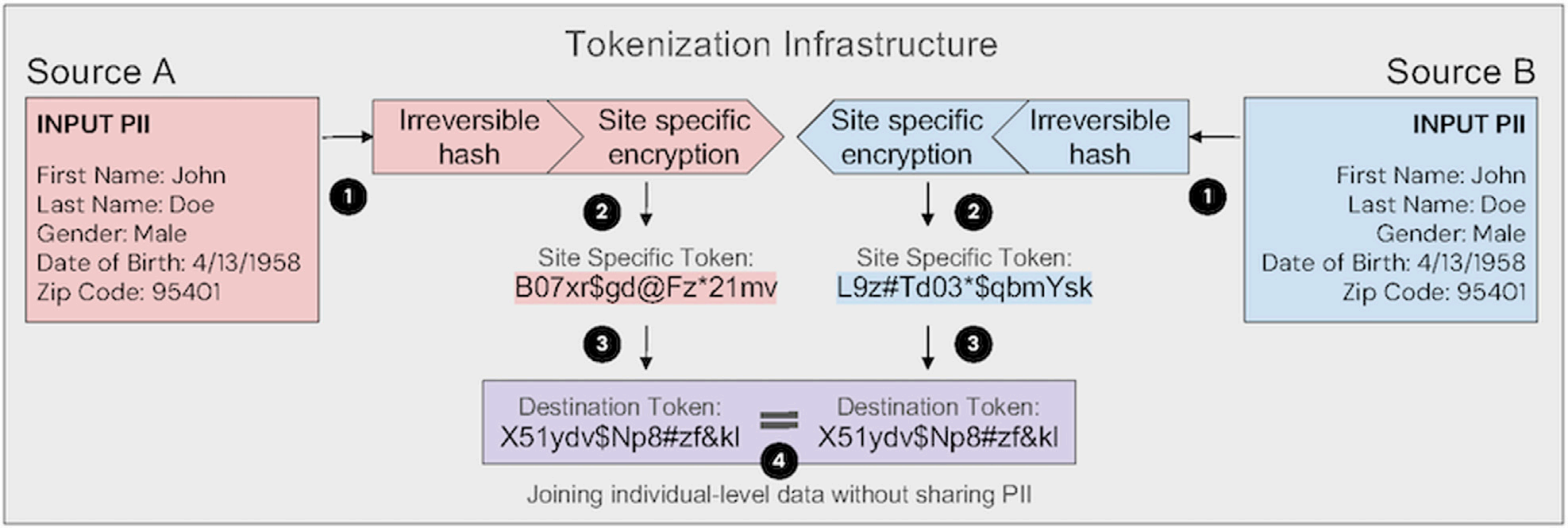
Tokenization infrastructure enabling privacy-preserving linkage across data sources. In step 1, input personally identifiable information (PII) is transformed using an irreversible hash. In step 2, the resulting “Master Token” is encrypted with a site-specific key, generating a unique site-specific token per source. This ensures that identical PII produces different tokens across organizations, enhancing security. In step 3, site-specific tokens are converted to a common destination token using standardized transformation protocols. In step 4, linked datasets are joined on destination tokens using predetermined matching algorithms, enabling individual-level data linkage without sharing PII. Expert attestation (also known as Expert Determination) under HIPAA is necessary to establish that data are de-identified and that reidentification risk is minimized.

As illustrated in Figure 1, token generation takes place at the source of the data, preserving privacy and minimizing the movement PII. Importantly, successful linkage via PPRL requires that all data contributors adopt the same underlying tokenization infrastructure; public and private systems are not interoperable by default. Selection between public and proprietary solutions may depend on factors such as governance models, technical support, scalability, and ease of integration with existing systems. Regardless of implementation, use of PPRL for secondary data analysis require an Expert Determination process to achieve de-identification under HIPAA standards. When properly implemented, token-based PPRL enables secure, longitudinal linkage of RWD such as claims, EHRs, and mortality records, supporting comprehensive pharmacovigilance in both pre- and post-market settings. Importantly, the utilization of any linkage methodology requires careful evaluation of internal and external validity and transparent reporting of the linkage and matching methods used (Pratt et al., 2020).

Centralized or federated data sharing models can be used with or without PPRL to access data sources in a privacy-preserving manner. In a centralized model using tokenization, the token generation is typically performed before data is transferred to a secure central environment. The linked, de-identified data is then housed centrally for downstream analysis, simplifying linkage logic and cohort curation, since all records are already co-located. In a federated model using tokenization, PPRL is used to generate shared tokens across sites while keeping identifiable data local. A data coordinating center, trusted third party or linkage honest broker may serve to send code to the federated sites or use tokens to pull matched cohorts, without revealing identities. Aggregate or model-level results can then be shared back centrally. The choice of data sharing setup depends on the use case, data sensitivity, partner preferences, and regulatory context.

### 4.3 Integration of PPRL into pre-market clinical trials and post-market surveillance

In the context of safety surveillance, PPRL can provide the ability to link EHRs and medical/pharmacy claims at the individual level to build a more complete picture of a potential safety signal or link across different claims and/or EHR databases over time to build a longitudinal journey of patients using different medical products. For clinical trials, this technology allows for the linkage of clinical trial participant data with RWD to fulfill use cases such as quantifying and mitigating data gaps due to lost to follow-up, long term real-world outcome assessment after completion of a trial, and collecting past medical history information that may not be reliable from self-report alone (Eckrote et al., 2024; Walters et al., 2025). For example, with PPRL a pharmaceutical company could link medical claims and mortality data for their trial cohort to better understand hospitalizations and mortality events in a real-world setting after the closure of a trial, all while ensuring that participant identities remain protected.

This linkage between trial participant and continued prospective evidence generation via RWD streamlines access to longitudinal patient data and accelerates the timeline for evidence generation. Instead of solely starting from scratch and having to wait months or years for patient data to accumulate from EHRs, insurance claims, or other disparate databases of patients that are completely different from those enrolled in the pre-market trials, continued follow-up of trial patients into their post-trial real-world medical product use provides an early evidence base on which to initiate safety surveillance.

### 4.4 Pre-market use of RWD: special considerations of reporting requirements

Regulatory frameworks established by agencies including the FDA, EMA, and International Council for Harmonisation (ICH) for the use of RWD and RWE in clinical trials and regulatory decision-making provide guidance for RWE stakeholders (U.S. Food and Drug Administration, 2018a; European Medicines Agency, 2025; European Medicines Agency, 2024). However, there are no explicit guidelines surrounding expectations and responsibilities when safety signals or events are detected in pre-market analyses using de-identified data. Here we outline considerations and what we consider to be best practices in this new space.

#### 4.4.1 Adverse event and safety signal reporting during clinical trials

To understand the full context of safety signal reporting and RWD, it is important to note that there are differences between long term post-market safety signal assessment (historically conducted using de-identified RWD) and what may be required as far as event reporting to an IRB for a prospective, pre-market trial. These differences include data type, traceability, and identification status.

#### 4.4.2 Regulatory and IRB reporting considerations for de-identified data

Good Clinical Practice (GCP) standards necessitate an expedited IRB/Independent Ethics Committee reporting process for all ADRs in clinical trials (ICH Harmonized Guideline for Good Clinical Practice E6(R3), 2025). If linked RWD are being used as part of long-term safety monitoring for a clinical trial, IRB and regulatory authority approval should be obtained before initiating the study. The sponsor’s pharmacovigilance and risk management experts should ensure procedures are aligned to their clinical development reporting requirements.

Plans should also be in place for reporting ADRs that come to the attention of study teams via de-identified analyses for purposes other than safety tracking (e.g., analyses of outcomes for endpoint reporting or HEOR studies). The protocol for the (non-safety) analysis should outline the plan for recording, evaluating, and reporting any incidental safety events or signals that are detected as part of the analysis process. It should also include explicit description of the de-identified nature of the analysis and if applicable, limitations regarding (in)ability for re-identification as may be the case if the RWD was obtained from a data aggregator and not a data originator or Covered Entity. The plan should be reviewed by the IRB and regulatory authorities before the study starts.

## 5 Discussion

Integrating tokenization and RWD linkage into the safety framework of clinical development offers a powerful toolset to enhance both pre- and post-market safety signal detection and monitoring. As discussed, tokenization enables privacy-preserving linkage of clinical trial data with de-identified RWD, allowing for earlier detection of unexpected safety concerns, such as chronic cardiovascular conditions, secondary malignancies, immune complications or neurologic signals uncovered through parallel healthcare resource utilization or other linked studies, prior to regulatory submission. Post-market, the same technologies can support long-term follow-up, especially in rare disease or novel therapy contexts such as cell and gene therapies, where the combination of de-identified RWD linkage and traditional medical record retrieval for deeper clinical investigation of AEs can facilitate comprehensive safety tracking over multi-year timeframes.

These approaches align with evolving regulatory expectations. Early engagement with regulatory agencies ensures that hybrid strategies maintain scientific rigor and meet requirements for safety reporting. Importantly, tokenization and linkage of RWD introduce specific considerations for regulatory reporting. Sponsors must clearly define, within safety and pharmacovigilance plans, which RWD-derived signals are considered reportable adverse events, particularly when data is de-identified and linked to clinical trial datasets and outline when and if re-identification is possible. These determinations are context-dependent and must be evaluated on a case-by-case basis.

Operationally, RWD-based safety strategies can help reduce costs by decreasing reliance on frequent site visits and by leveraging existing healthcare data. This model is especially beneficial for long-term studies, where shifting to passive follow-up strategies via linked RWD minimizes participant burden, supports higher retention, and increases data completeness. In parallel, utilizing linked RWD enables monitoring across diverse populations and care settings, helping bridge data gaps when participants are lost to follow-up, when long-term outcomes extend beyond the trial infrastructure, or when populations of interest were not included in clinical trials.

Nonetheless, implementing PPRL for pharmacovigilance across pre- and post-market settings requires careful attention to both operational and methodological challenges. Data quality, interoperability, and careful data transformation are foundational, as inconsistent identifiers and heterogeneous data structures can compromise linkage accuracy and downstream analyses; standardized preprocessing and harmonization of data elements are critical. Matching algorithms must appropriately balance precision (positive predictive value) and recall (sensitivity), with validation studies needed to assess matching accuracy as well as uncover biases in population representation. Clear outcome and endpoint definitions and methodological consistency across data sources are essential to ensure valid signal detection and interpretation. Privacy and compliance must be maintained through privacy-by-design approaches, robust encryption, and well-defined data use agreements. Furthermore, effective consent management and stakeholder coordination, among sponsors, clinical research sites, data partners, and regulators, are crucial for implementation at scale. Finally, transparency in linkage methods, auditability of the process, and alignment with regulatory expectations help ensure that linked real-world and clinical trial data can be confidently used for safety monitoring across the product lifecycle.

Overall, integrating tokenization and RWD linkage into safety signal assessment can create a robust, cost-effective, and scalable model for modern drug development. These innovations enable proactive safety surveillance, strengthen regulatory engagement, and ultimately enhance the real-world applicability and trust in emerging therapies. As the field evolves, ongoing refinement of regulatory guidance and best practices will be essential to fully realize the potential of these approaches.

## Data Availability

The original contributions presented in the study are included in the article/Supplementary Material, further inquiries can be directed to the corresponding author.
