# Correction: Leveraging real-world data for safety signal detection and risk management in pre- and post-market settings

**DOI:** 10.3389/fdsfr.2025.1729828

**Published:** 2025-11-07

**Authors:** Kathleen M. Gavin, Matthew L. Sundermann, Alethea Wieland

**Affiliations:** 1 Datavant, Phoenix, AZ, United States; 2 Advarra, Columbia, MD, United States

**Keywords:** real world data, safety surveillance, pharmacovigilance, risk management, privacy preserving record linkage (PPRL), tokenization, data linkage, federated data sharing

The reference for [EMA, 2017] was erroneously written as “EMA (2017). Guideline on good pharmacovigilance practices (GVP) module IX–Signal management. Available online at: https://www.ema.europa.eu/en/documents/scientific-guideline/guideline-good-pharmacovigilance-practices-gvp-module-ix-signal-management-rev-1_en.pdf.” It should be “European Medicines Agency (2017). Guideline on good pharmacovigilance practices (GVP) module IX–Signal management. Available online at: https://www.ema.europa.eu/en/documents/scientific-guideline/guideline-good-pharmacovigilance-practices-gvp-module-ix-signal-management-rev-1_en.pdf”.

The reference for European Medicines Society, 2023 was erroneously written as “European Medicines Society (2023). ICH M14 general principles on plan, design and analysis of pharmacoepidemiological studies that utilize real-world data for safety assessment of medicines - scientific guideline. Available online at: https://www.ema.europa.eu/en/ich-m14-guideline-general-principles-plan-design-analysis-pharmacoepidemiological-studies-utilize-real-world-data-safety-assessment-medicines-scientific-guideline”. It should be “European Medicines Agency (2025). ICH M14 general principles on plan, design and analysis of pharmacoepidemiological studies that utilize real-world data for safety assessment of medicines - scientific guideline. Available online at: https://www.ema.europa.eu/en/ich-m14-guideline-general-principles-plan-design-analysis-pharmacoepidemiological-studies-utilize-real-world-data-safety-assessment-medicines-scientific-guideline”.

The reference for European Medicines Society, 2025 was erroneously written as “European Medicines Society (2025). Data analysis and real world interrogation network (DARWIN EU). Available online at: https://www.ema.europa.eu/en/about-us/how-we-work/data-regulation-big-data-other-sources/real-world-evidence/data-analysis-real-world-interrogation-network-darwin-eu”. It should be “European Medicines Agency (2025). Data analysis and real world interrogation network (DARWIN EU). Available online at: https://www.ema.europa.eu/en/about-us/how-we-work/data-regulation-big-data-other-sources/real-world-evidence/data-analysis-real-world-interrogation-network-darwin-eu”.

The reference for Rosati et al., 2022 was erroneously written as “Rosati, K., Jorgensen, N., Soliz, M., and Evans, B. (2022). HIPAA and common rule compliance in the Sentinel initiative. Sentinel initiative principles and policies”. It should be “Rosati, K., Jorgensen, N., Soliz, M., and Evans, B. (2018). HIPAA and common rule compliance in the Sentinel initiative. Sentinel initiative principles and policies. Available at: https://www.sentinelinitiative.org/sites/default/files/communications/publications-presentations/HIPAA-Common-Rule-Compliance-in-Sentinel-Initiative.pdf”.

The reference for U.S. Food and Drug Administration, 2023a was erroneously written as “U.S. Food and Drug Administration (2023a). Data standards for drug and biological product submissions containing real-world data guidance document. Available online at: https://www.fda.gov/regulatory-information/search-fda-guidance-documents/considerations-use-real-world-data-and-real-world-evidence-support-regulatory-decision-making-drug”. It should be “U.S. Food and Drug Administration (2023a). Data standards for drug and biological product submissions containing real-world data guidance document. Available online at: https://www.fda.gov/regulatory-information/search-fda-guidance-documents/data-standards-drug-and-biological-product-submissions-containing-real-world-data”.

The original article has been updated.

