# Supplementary material for: Leveraging real-world data for safety signal detection and risk management in pre- and post-market settings
Source: Front Drug Saf Regul. 2025 Oct 17;5:1626822. doi: 10.3389/fdsfr.2025.1626822 (PMC12576344; doi:10.3389/fdsfr.2025.1626822)
Supplement: Supplementary file 1 [file Supplementaryfile1.docx]

Leveraging Real-World Data for Safety Signal Detection and Risk Management in Pre- and Post-Market Settings

Kathleen M Gavin, PhD,^1^* Matthew L Sundermann, PhD,^2^ Alethea Wieland^2^

^1^Datavant, Phoenix, AZ, USA

^2^Advarra, Columbia, MD, USA

Supplementary Material

# Common Terms and Definitions

There are various terms describing processes for safety monitoring in the pre- and post-market settings. A few of these terms are highlighted in Table 1.

Table 1: Terms and Definitions for Post-Market Surveillance

| Term | Definition |
| --- | --- |
| Adverse Drug Reaction (ADR) | An adverse drug reaction (ADR) can be defined as ‘an appreciably harmful or unpleasant reaction resulting from an intervention related to the use of a medicinal product’([1](#_ENREF_1)) |
| Adverse Event (AE) / Serious Adverse Event (SAE) | An adverse event (AE) is any undesirable experience associated with the use of a medical product in a patient. An AE is serious (SAE) if it is life threatening, involves a hospitalization, is associated with disability or permanent damage, is associated with birth defects, or is associated with other serious medical events ([2](#_ENREF_2)) |
| Development Safety Update Report (DSUR) | The development safety update report (DSUR) is a periodic report on drugs under development among the ICH regions. These drugs include marketed drugs that are under further study ([3](#_ENREF_3)). |
| Pharmacovigilance | The science and activities relating to the detection, assessment, understanding and prevention of adverse effects or any other medicine/vaccine related problem ([4](#_ENREF_4)). |
| Risk Management Plan | The Risk Management Plan includes information on a medicine’s safety profile, risk prevention strategies in patients, plans for studies on safety and efficacy of the medicine, and measures on how to measure the effectiveness of risk minimization ([5](#_ENREF_5)). |
| Safety Management Plan | The Safety Management Plan is a component of pharmacovigilance that covers procedures and processes for managing potential safety risks including identification and assessment of safety risks ([6](#_ENREF_6)). |
| Safety Signal | Reported information on a possible causal relationship  between an adverse event and a [medical product], the relationship being previously unknown or incompletely documented ([4](#_ENREF_4)). |
| Safety Surveillance | The science of detecting, assessing, and preventing issues related to medical products. |
| Signal Management | A component of pharmacovigilance involving the gathering of necessary information required to support the benefit-risk profile and ensure the safety of a pharmaceutical or biological product ([7](#_ENREF_7)). |
| Suspected Unexpected Serious Adverse Reaction (SUSAR) | A suspected unexpected serious adverse reaction (SUSAR) is an adverse drug reaction that is unexpected, and suspected to be caused by a medicinal product. |

# Checklists

Users of de-identified RWD for clinical evidence generation programs (both pre-market and post-market) may benefit from the following best practices checklists. These checklists serve as a guide to help align with regulatory acceptability, assure that all ADR reporting requirements are addressed, and strengthen the post-market Risk Management Plan of the sponsor. Note, not all parts of the checklists will be applicable to all use cases but are to serve as a general guideline for what may be needed. There may be other regulatory or compliance requirements that are not included here. It is up to the sponsor to ensure they are following all regulatory and compliance guidelines that apply to their specific post-market surveillance or post-market commitment programs.

## Checklist 1 - Key Considerations for Planning and Executing Studies Utilizing Tokenization, RWD Linkage, and Analysis

Key study elements that need to be planned for in studies utilizing tokenization, RWD linkage and analysis of RWD including the following:

Note, this list is non-exhaustive and represents key elements related to tokenization and linking RWD. See FDA guidance, Real-World Data: Assessing Electronic EHR and Medical Claims Data to Support Regulatory Decision Making for Drug and Biological Products for a full description of protocol considerations ([8](#_ENREF_8)).

### Protocol(s)

1. Data Source Selection:
   1. Justification of the selected data sources for answering study questions.
   2. Evaluation of the relevance, reliability, and potential limitations of these data sources in accurately capturing exposures, outcomes, and covariates.
      1. Framework for assessing data quality and potential biases.
2. Data Tokenization and Linkage Methodology:
   1. Detailed description of Personally Identifiable Information (PII) collection methods and quality and token generation methodology.
   2. Detailed description of data linkage/matching methods (e.g., probabilistic or deterministic) and/or algorithms.
   3. Validation studies of linkage quality and methods including the accuracy and reliability of matching process and addressing linkage errors or discrepancies.
3. Study Design Elements:
   1. Time Periods: Clear definition of time frames for exposure, outcome assessment, and follow-up.
   2. Population Selection: Clinical and operational definitions for inclusion/exclusion criteria to identify eligible patients from linked data.
      1. Inclusion and exclusion criteria for patient populations are defined. This section specifies the demographic, clinical, and geographic characteristics of patients to be included in the analysis, ensuring alignment with the product’s approved indication and study objectives.
4. Adverse Drug Reactions (ADRs)
   1. This section specifies how adverse drug reactions will be identified, categorized, and reported, including both expected and unexpected events.
5. Exposure Ascertainment and Validation: Definitions of exposures (e.g., dose, duration), methods for validation, and considerations for missing or incomplete exposure data.
   1. The protocol details how the duration of medical product administration will be captured and analyzed, including patterns of use and potential deviations from approved indications.
6. Outcome Definitions and Validation: Conceptual and operational definitions for outcomes, including approaches for validation to minimize misclassification.
   1. The primary and secondary endpoints will be detailed. Definitions for endpoints will follow established clinical guidelines and standards, including both clinical and operational definitions as described below.
      - 1. Conceptual definition: including study population inclusion and exclusion criteria, exposure, outcome, and covariates. A conceptual definition should reflect current medical and scientific thinking regarding the variable of interest, such as: (1) clinical criteria to define a condition for population selection or as an outcome of interest or a covariate; or (2) measurement of drug intake to define an exposure of interest.
        2. Operational Definition: operational definition should be developed based on the conceptual definition to extract the most complete and accurate data from the data source. In many studies using EHRs or medical claims data, the operational definition will be a code-based electronic algorithm using structured data elements. In other studies, the operational definition may be derived from extracting relevant information from unstructured data or constructing an algorithm that combines structured and unstructured data elements
7. Covariates: Identification of confounders and effect modifiers, along with methods to measure or validate these within the data sources.
8. Data Quality Assurance:
   1. Processes for data accrual, including traceability to original sources, handling of discrepancies, and duplicate records.
   2. Quality control (QA/QC) plan detailing the steps for ensuring data integrity, accuracy, and completeness during data accrual, curation, and transformation.
9. Handling Missing Data:
   1. Identification of missing data scenarios and their potential impact on study validity.
   2. Strategies for addressing missing data through data linkage, proxies, or sensitivity analysis.
10. Documentation and Reporting:
    1. Detailed documentation of data management processes, linkage, transformation methods, and adherence to the protocol and analysis plan.
    2. Justification of any data standardization or modifications and their impact on the study's validity.
    3. Detailed description of the (in)ability to perform re-identification on the participants in the real-world data.
11. Considerations for Exempt Research Determination
    1. This section will evaluate whether the study qualifies as exempt research based on applicable ethical guidelines, with a focus on the use of de-identified data and the minimal risk to participants.

### Data Management Plan (DMP)

1. The data management plan defines the secure handling, storage, and analysis of trial and linked RWD, with a focus on data privacy, traceability, quality assurance, and audit trails. The DMP should include a framework for the linking strategies, including matching strategies to be used, plan for meeting HIPAA de-identification standards, as well as QC/QA steps outlined for reliable PII entry, how to handle data entry errors, and adjudicate duplicate entries, changes in PII over time, and withdrawal of tokens for participants who withdraw their consent.
2. Procedures for data acquisition, cleaning, and processing should be be outlined. This includes how data will be de-identified according to HIPAA standards, linked, and stored securely to ensure compliance with regulatory and privacy standards.

### Statistical Analysis Plan (SAP)

1. Specifies the statistical methods and endpoints for the analyses. Analysis methods account for potential confounders and bias, particularly when interpreting real-world data that may reflect biases related to real-world treatment patterns and co-morbidities and availability of data.
2. Includes detailed methodologies for handling missing data and conducting sensitivity analyses. Statistical models, subgroup analyses, and pre-specified hypotheses should be clearly described.

### Safety Management Plan

1. A comprehensive safety management plan addresses signal detection, evaluation, and reporting obligations. It includes formalized standard operating procedures (SOPs) for identifying, assessing, and reporting potential new safety signals identified through linked RWD. A safety management plan should consider the potential for safety signals that could be revealed in analyses of de-identified RWD and any identifiable RWD workflows and how those findings will be reported and integrated into the greater safety management plan for the clinical program. Roles, responsibilities, and escalation pathways should be specified.

### Investigator Brochure Updates

1. As new safety and efficacy data from RWD analyses become available, updates to the investigator brochure may be warranted to inform clinical investigators of emerging risks and findings.

### Development Safety Update Reports (DSURs)

1. Annual DSURs integrate safety data from both clinical trials and other sources, summarizing cumulative safety data and any new safety signals identified during the reporting period. Findings from RWD studies should be integrated in the DSURs as applicable.

### Clinical Study Report (CSR)

1. The CSR includes a comprehensive summary of the clinical trial or post-approval safety study (PASS) outcomes. Depending on the timing and integration of RWD studies to study protocol, RWE may not be part of the CSR, but rather summarized in a separate report to be utilized as applicable by the sponsor in supporting clinical decision making or payor evidence packages. How RWE is integrated into the CSR depends on how it is utilized in relation to the study. The CSR should be prepared in accordance with regulatory guidelines and submitted to relevant authorities.

### Risk Assessment of Re-Identification

1. A comprehensive risk assessment evaluates the potential for re-identification of de-identified data, including an analysis of residual risks and mitigation strategies such as tokenization and aggregation.
2. The procedures to mitigate the risk of re-identification should be described, emphasizing the use of expert statistical review to address HIPAA compliance and adherence to applicable privacy laws. Safety events detected in de-identified data sources will likely need to be reported in a de-identified fashion without the ability to re-identify.

### Other Post-Market Activities for Customer and Rest-of-World Complaints

1. Procedures for addressing customer complaints and ADRs reported outside the study region should be described, including integration into global safety monitoring systems.

### Competitor Safety Information

1. This describes how safety information from competitors’ products will be monitored, evaluated, and contextualized to enhance the understanding of the medical product's safety profile.

### Overall Risk Management Plan Implementation and Management

1. The overarching risk management plan (RMP) will integrate findings from the PASS and other safety studies, outlining strategies for mitigating identified risks and communicating them to stakeholders.

### Literature Reviews

1. Regular reviews of the scientific literature are conducted to identify emerging safety signals, contextualize study findings, and ensure the PASS remains aligned with current evidence.

##

## Checklist 2 - Tokenization Implementation Considerations

This checklist outlines critical elements to consider when planning and implementing tokenization and RWD linkage in clinical studies. To ensure compliance with regulatory expectations and ethical guidelines, sponsors must address specific requirements in their clinical protocols, informed consent forms (ICF), and operational processes.

The checklist below provides an outline to help confirm alignment with FDA guidance, ensure clarity in patient-facing materials, and establish robust operational workflows for tokenization and data linkage. These considerations are designed to support effective, compliant, and patient-centric integration of tokenization into clinical research programs.

1. Engage with FDA to ensure (planned) use of RWD is aligned with evidence requirements
2. Confirm that clinical protocol describes:
   - Token Generation
   - Token Matching and Data Linkage Process
   - Use cases for linked RWD including types of RWD if applicable
   - De-identification considerations (description of whether identified individual level reporting will be possible or not)
   - Safety monitoring plan including plan for any events detected in de-identified RWD as part of explicit safety, or any other, analysis of the data.

Note: See Checklist 1 for more detailed protocol considerations

1. Confirm that the clinical protocol aligns with FDA Guidance on Real-World Data: Assessing Electronic Health Records and Medical Claims Data to Support Regulatory Decision-Making for Drug and Biological Products ([8](#_ENREF_8))
2. Confirm that language in the ICF reflects both token generation and potential future linkage to RWD. This includes:
   - Risks and benefits of tokenization and linking RWD
   - Opportunity for withdrawal of consent
   - Time duration for which tokens will be stored/utilized
   - Use cases within scope for data linkage with their tokens (e.g., within a specific therapeutic area, for a specific medical product, future research activitites)
3. Stakeholder review of protocol and ICF
4. Have all protocols, ICF and patient facing educational materials reviewed and approved by IRB and FDA, as applicable.
5. Confirm token generation as part of clinical operations process – outline the overall data flow, determine what PII will be collected, where that PII is located or stored, how tokens will be generated, where tokens will be stored, and how quality controls will be implemented.

## Checklist 3 - Safety Reporting Structure

A well-defined safety reporting structure is essential for ensuring timely and accurate communication of ADRs and safety signals whether events are detected as part of a clinical trial or postmarketing program. This checklist provides a framework for establishing a safety reporting process that aligns with regulatory requirements, internal standard operating procedures (SOPs), and clinical protocols. By following these guidelines as a starting point, sponsors can build a transparent and effective safety management system that supports patient safety and regulatory obligations.

1. Consult w/ Relevant Stakeholders to formulate safety reporting structure
   - Pharmacovigilance
   - Legal
   - Clinical and Medical Affairs
   - Regulatory Affairs
2. Confirm ADR reporting procedures in clinical protocol
   - Evaluate involved data partners and identify which are strictly de-identified and which can work in an identified workflow for reporting purposes
   - Detail the required reporting procedures for identified data vs de-identified data
3. Ensure the reporting process conforms to relevant internal SOPs or clinical protocol or update them as necessary.
4. Stakeholder review of safety reporting plan
5. Have reporting plan/SOP reviewed and approved by IRB and FDA as applicable
6. When ADR or safety signals are detected, the study staff of the analytics team follows SOP for reporting requirements.

#

# References

1. Aronson JK, Ferner RE. Clarification of terminology in drug safety. Drug safety. 2005;28:851-70.

2. What is a Serious Adverse Event: US FDA; [Available from: <https://www.fda.gov/safety/reporting-serious-problems-fda/what-serious-adverse-event>.

3. ICH Harmonized Tripartite Guideline: Development Safety Update Report E2F, (2010).

4. WHO Pharmacovigilance Indicators: A Practical Manual for the Assessment of Pharmacovigilance Systems. World Health Organization; 2015.

5. Risk Management Plans [Available from: <https://www.ema.europa.eu/en/human-regulatory-overview/marketing-authorisation/pharmacovigilance-marketing-authorisation/risk-management/risk-management-plans>.

6. Developing the Safety Management Plan (SMP BIOMAPAS [Available from: <https://www.biomapas.com/safety-management-plan-smp-pharmacovigilance/>.

7. Beninger P. Signal Management in Pharmacovigilance: A Review of Activities and Case Studies. Clinical Therapeutics. 2020;42(6):1110-29.

8. Real-World Data: Assessing Electronic Health Records and Medical Claims Data to Support Regulatory Decision-Making for Drug and Biological Products, (2024).
